# Umbilicus: A Site for Stoma in Hirschsprung’s Disease

**Published:** 2015-04-01

**Authors:** Prince Raj, Yogesh Kumar Sarin

**Affiliations:** Department of Pediatric Surgery, Maulana Azad Medical College, and associated Lok Nayak Hospital, New Delhi

A 13-day-old male baby with the symptoms suggestive of HD and barium enema showing the transition zone (TZ) at the recto-sigmoid was admitted with progressive abdominal distention not responding to rectal washes. The baby was planned for sigmoid colostomy and seromuscular biopsy. A transverse skin incision of 2cms was made through the umbilicus under general anesthesia. The skin, subcutaneous tissue, and fascia were incised, and the umbilical vessels and urachal remnant were individually ligated apart from the opening in the fascia. At this point, a urethral sound was passed per rectally to guide the sigmoid loop towards the umbilicus. Stay sutures were taken and the loop of sigmoid colon was taken out. Biopsies were taken from the recto-sigmoid junction and site proximal to TZ. The bowel wall was fixed separately to the peritoneum and fascia with interrupted 5-0 absorbable sutures. The bowel was opened and everted with suturing to the skin (Fig. 1).

**Figure:**
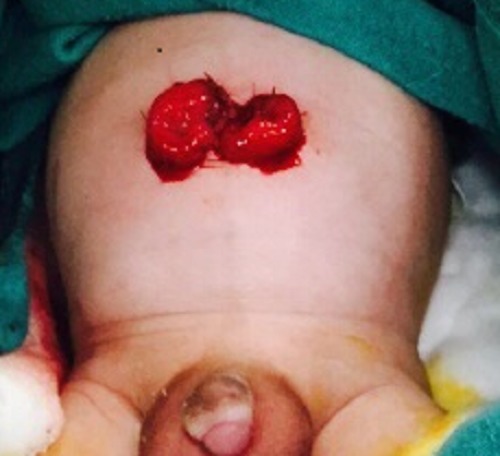
Figure 1: Umbilical divided colostomy

Management of Hirschsprung’s disease (HD) has evolved in last one decade from staged procedure to single stage trans-anal pull through. But in developing country like ours, the presentation is generally late (most of them brought with severely dilated colon or enterocolitis) and where there is lack of facility for frozen section biopsy, staged procedures are still the standard of care. In this era of minimal access surgery, where a lot of stress is laid on cosmesis, role of umbilical incision for fashioning of colostomy and taking biopsies is a good alternative. Though trans-umbilical colostomies have been occasionally done in past for HD and anorectal malformations1,2,3, they are still not widely used. 

This technique has several advantages:

1. Less time consuming, cost effective and easy to perform.
2. Provides easy access to both the sigmoid and transverse colon.
3. Umbilicus lies on the center of the abdomen and hence the stoma bag can be well placed and stoma care will be easy.
4. Better cosmesis as the scar will not be visible once the stoma is closed.
5. Does not require advanced surgical skill as in laparoscopy.
6. Delayed trans-anal pull through can still be performed through the same incision.

In today’s world smitten with minimal accesses surgery, this approach will serve as an attractive alternative in the staged management of HD, especially in a case of a peripheral center that may lack or cannot afford laparoscopy.

## Footnotes

**Source of Support:** Nil

**Conflict of Interest:** The author is editor of the journal but he is not involved in decision making of this manuscript.

